# Preconditioning with SDF-1 Improves Therapeutic Outcomes of Bone marrow-derived Mesenchymal Stromal Cells in a Mouse Model of STZ-induced Diabetes

**Published:** 2019

**Authors:** Mohammad Sadegh Gholami Farashah, Parichehr Pasbakhsh, Ameneh Omidi, Saied Nekoonam, Roya Aryanpour, Iraj Regardi Kashani

**Affiliations:** 1. Department of Anatomical Sciences, Faculty of Medicine, Tehran University of Medical Sciences, Tehran, Iran; 2. Department of Anatomical Sciences, Faculty of Medical Sciences, Tarbiat Modares University, Tehran, Iran

**Keywords:** Bone marrow-derived mesenchymal stromal cells, Diabetes mellitus, Homing, SDF-1

## Abstract

**Background:**

Nowadays, transplantation of Bone marrow-derived Mesenchymal Stromal Cells (BMSCs) is currently an important alternative therapy for patient’s type 1 diabetes mellitus. But a number of critical obstacles lie ahead of this new strategy including reducing stem cell homing to the damaged tissue due to oxidative stress. The purpose of the present study was to investigate whether preconditioning of BMSCs with SDF-1 could enhance their homing to the pancreas and promote regeneration of the pancreatic β cells after being intravenously injected.

**Methods:**

Mice BMSCs were isolated and expanded. Cell proliferation was assayed by MTT Assay. Preconditioning was performed with 10 *ng/ml* SDF-1α for 24 *hr*. Male NMRI mice were injected with high-dose STZ (150 *mg/kg*). The preconditioned or un-preconditioned BMSCs at a dose of 1×10^6^ cells were infused via the tail vein. Blood and pancreatic tissue samples were taken from all mice for flow cytometry, biochemical and histological studies.

**Results:**

Proliferation and homing of BMSCs to the pancreas were significantly increased in the BMSCs with SDF-1α preconditioning. Differentiation of transplanted BMSCs, were significantly increased in preconditioning group. Although BMSCs without SDF-1 preconditioning exhibited remarkable recovery of pancreatic islets structure but this recovery were significantly increased in the BMSCs with SDF-1α preconditioning.

**Conclusion:**

Our results showed the effectiveness of SDF-1α preconditioning in BMSCs transplantation of STZ induced diabetes mice which might be achieved through improvement of BMSCs homing into the injured pancreas.

## Introduction

Type 1 diabetes mellitus is a chronic autoimmune disease with a strong inflammatory component that is diagnosed in childhood and requires a lifetime of self-management at home and in the community between regular consultations with the health care team 1. Type 1 diabetes mellitus pathophysiology seems to consist of a direct destruction of pancreatic β-cells through auto antibodies against beta-cells 2.

While there are promising therapeutics in the drug pipeline (*e.g*. anti-CD3), there are currently no treatments that arrest or reverse progressive destruction of insulin-producing beta cells 3. To date, pancreatic islet transplantation is an emerging therapeutic option for diabetic patients 4. But this technique has serious limitation due to the shortage of cadaveric islet donors 5. Fortunately, in response to this requirement many researchers have developed novel experimental strategies to generate β-cells by transplantation of stem cells from different sources including embryos and adult tissues 6.

Bone marrow-derived Mesenchymal Stromal Cells (BMSCs) may be the ideal source of cells as they are easily purified and allow auto grafts to be performed 7. Numerous experimental studies have suggested that BMSCs ameliorate diabetes in animal models. Successful clinical outcomes from transplantation of BMSCs depend upon efficient BMSCs homing into the damaged area 8.

But a number of critical obstacles lie ahead of this new strategy including reducing stem cell homing to the damaged tissue due to oxidative stress, inflammation and hypoxia, apoptotic cascade activation, poor vascular supply, insufficient trophic factors and loss of survival factor in injured area when delivered by local or systemic routes 9. A number of studies have shown that the BMSCs migrate to sites of injury and inflammation in response to soluble mediators including the chemokine stromal cell-derived factor (SDF-1), but during *in vitro* culture expansion BMSCs lose surface expression of key promoting homing receptors particularly SDF-1α receptor, cognate receptor CXC chemokine receptor 4 (CXCR4) 10. This may be due to decrease in the response ability of the transplanted cells to homing signals originating from the injured area 11. In order to compensate the mentioned loss in receptors, in the present study, BMSCs were pretreated with SDF-1. SDF-1, also called CXCL-12, is a key regulatory chemokine secreted by MSCs 12. SDF-1/CXCR4 signaling cascade increases growth and survival of BMSCs 13. The purpose of the present study was to investigate whether preconditioning of BMSCs with SDF-1 could enhance their homing to the pancreas and promote regeneration of the pancreatic β cells after being intravenously injected.

## Materials and Methods

### Ethics statement

All experiments were approved by the Committee on the Ethics of Animal Experiments of the Tehran University of Medical Sciences, Iran (Permit Number: IR.TUMS.MEDICINE.REC.1395.1001). All surgery was performed under anesthesia, and all efforts were made to minimize suffering.

### Isolation and culture of BMSCs

BMSCs were harvested from bone marrow of the femurs and tibias of 4–6 week-old male mice obtained from Pasteur Institute, by inserting a 21-gauge needle into the shaft of the bone and flushing it with 10 *ml* of Dulbecco’s modified Eagle’s medium supplemented with 1000 *U* heparin (Roche Pharma, Switzerland). The cell suspension was centrifuged over a Ficoll step gradient (density 1.067 *g/ml*) (Sigma, St. Louis, MO) at 1500 *rpm* for 10 *min*. The interface fraction was then collected and cultured in DMEM, supplemented with 10% fetal bovine serum (FBS, GIBCO, USA) and 1% penicillin/streptomycin (pen/strep, Invitrogen) solution. Isolated cells were grown at 37°*C* and 5% CO_2_ for 3 days. After removing suspension cells, the adherent BMSCs were grown to 90% confluence and used between passages 1 and 3. For characterization of BMSCs, BMSCs were analyzed by fluorescence activated cell sorting (FACS). Cells in the fourth passage were incubated in primary antibodies as follows: in mouse and rat monoclonal CD45(1:100, BD Biosciences, USA), mouse monoclonal CD34 (1:100 BD Biosciences, USA), mouse monoclonal CD73 (1:100, BD Biosciences, USA) and mouse monoclonal CD90 (1:100 BD Biosciences, USA) antibodies for 40 *min* at 4°*C*. Then, the cells were incubated with secondary antibodies for 30 *min* at room temperature. Secondary antibody for CD34 (1: 200 Abcam, UK) was conjugated with phycoerythrin, while secondary antibody for D45, CD73 and CD90 (1: 100 Abcam, UK) were conjugated with fluorescein isothiocyanate. The mouse IgG_1_ (Dako) was used for the isotype control.

### Cell proliferation assay (MTT)

Cell proliferation of BMSCs was assayed by measuring MTT Cell Proliferation Assay (Roche Applied Science, Indianapolis, USA), an *in vitro* assay for the measurement of formazan produced from MTT by dehydrogenase of mitochondria, which is active merely in live cells. Initially, seeded BMSCs, 10^4^ per well in 96-well micro-plates (Nunc, Roskilde, Denmark), were incubated for 24 *hr*. The medium was then replaced with serum-free DMEM containing 10 *ng/ml* SDF-1α for another 1 day. The assay was performed with Cell Proliferation Kit I (MTT; Boehringer Mannheim, Mannheim, Germany).

### Preconditioning of BMSCs by SDF-1α

To determine the effect of SDF-1α (SDF-1α, Sigma, USA) on BMSCs, cells were subjected to a 1day preconditioning with 10 *ng/ml* SDF-1α 14. SDF-1α-preconditioned BMSCs (SDF-BMSCs) were then washed three times with PBS (Sigma, USA) for complete removal of the factor from cell suspension.

### Induction of type 1 diabetes mellitus with STZ and cell transplantation

Type 1 diabetes mellitus model in mice was established as described previously 15. Briefly, streptozotocin (STZ) was administered by high single dose intraperitoneal injection (150 *mg/kg* in 50 *mM* Sodium citrate; pH=4.5) in eight week old male NMRI mice. Blood glucose levels were determined in blood samples from the tail vein using glucometer system (Bionime glucometer, model GM110). Mice were considered diabetic when glycemia exceeded 250 *mg/dl* in two consecutive determinations. In the same day, Body Weights (BW) of the diabetic mice were measured and recorded. Groups were as follows: Diabetic+PBS (Diabetic); diabetic+unpreconditioned BMSCs (Diab+ BM-SCs); diabetic+SDF-1α preconditioned BMSCs (Diab+BMSCs+SDF-1α). Prior to transplantation, BMSCs were labeled with chloromethylben-zamido-1,1′-dioctadecyl-3,3,3′,3′ tetramethylindo-carbocyanine perchlorate (CM-DiI; 1 *μg/ml*; Invitrogen, USA) at 37°*C* for 30 *min*. Several studies have demonstrated that labeling cells with CM-DiI does not affect cell viability, proliferation or differentiation 16. At week 4 after STZ induction of diabetes, preconditioned (BMSCs+SDF-1α) or unpreconditioned BMSCs (CM-DiI-labeled) at a dose of 1×10^6^ cells/0.2 *ml* PBS were infused *via* the tail vein. The same volume of PBS (0.2 *ml*) was injected as a vehicle.

### Detection of CM-DiI positive BMSCs

Forty eight hours after cell administration, male mice were anesthetized with intraperitoneal injection of ketamine and xylazine mixture (ketamine 120 *mg/ml*, 1.0 *ml* and xylazine 20 *mg/ml*, 0.5 *ml*), perfused transcardially with 4% paraformaldehyde (Sigma, USA) in 0.1 *mol/L* phosphate buffer (PB; pH=7.2), and the organs of mice post-fixed in the same oxidative overnight. Samples were cut into 6 *μm* sections and were stained with 4’, 6-diamino-2-phenylindole (DAPI; Sigma, USA). Quantification of fluorescence intensity of positive cells was done using ImageJ software (Image J for Windows, Version 1.50i) 6.

### Implantation analysis by flow cytometry

Forty eight hours after transplantation, NMRI mice were perfused with normal saline, and tissue samples were gently smashed by forceps and placed in a tube containing collagenase IV (Invitrogen, USA). The suspension was passed through 150 *μm* mesh and centrifuged (200×*g*, 5 *min*). The mononuclear cells were obtained from the pancreas and other tissues; the cells were procured in PBS, washed, subjected to red blood cell lysis, and suspended in PBS containing 1% paraformaldehyde for analysis of fluorescence intensity using flow cytometry. The samples were carried out in triplets and evaluated for red fluorescence CM-DiI at 570 *nm*. Fluorescence intensity was quantified using Image J software (Image J for Windows, Version 1.50i; NIH, Bethesda, MD, USA; available in the public domain at http://rsb.info.nih.gov/ij/index.html).

For this purpose, then, it was exposed to 1 *ml* lysis buffer and finally suspended in 1 *ml* PBS containing 1% paraformaldehyde for further analysis of fluorescence intensity by flow cytometry. The samples were carried out in triplets and evaluated for red fluorescence CM-DiI at 570 *nm*.

### Histological and immunohistochemical analysis

30 days after cell transplantation, firstly, bodyweights of all animals were recorded, and then pancreatic samples were fixed with formalin, and embedded in paraffin. For histological examinations, cross-sections (5 *μm*) were stained with Hematoxylin and Eosin (H&E). For immunohistochemical analyses, sections were permeabilized with 0.2% Triton X-100 (Sigma, USA) in PBS for 30 *min*, blocked with 3% Bovine Serum Albumin (BSA) in PBS for 1 *hr*, and incubated with anti-insulin antibody (1:100; Abcam, UK). After washing in PBS (pH=7.4), slides were incubated with FITC-conjugated secondary antibody (1:200; Abcam, UK) at 4*°C* for 1 *hr* and stained with DAPI dye for 30 *min* at room temperature under dark conditions to label the nuclei with fluorescent. Cells were counted in three nonadjacent 7 *μm* longitudinal sections per pancreas (n=3/group). Insulin positive (Insulin+) and CM-DiI positive with insulin positive (CM-DiI+/Insulin+) cells were counted under a fluorescence microscope (BX51; Olympus, Tokyo, Japan) using a 40× objective and a grid overlay. Average cell densities were calculated as number of cells per *mm*^2^.

### Statistical analysis

Data are presented as mean±standard deviation (SD). Statistical comparisons included unpaired/paired t tests or one-way analysis of variance with Turkey’s post-test. p<0.05 was considered significant.

## Results

### Characterization of BMSCs in in vitro culture

**Figure 1.**
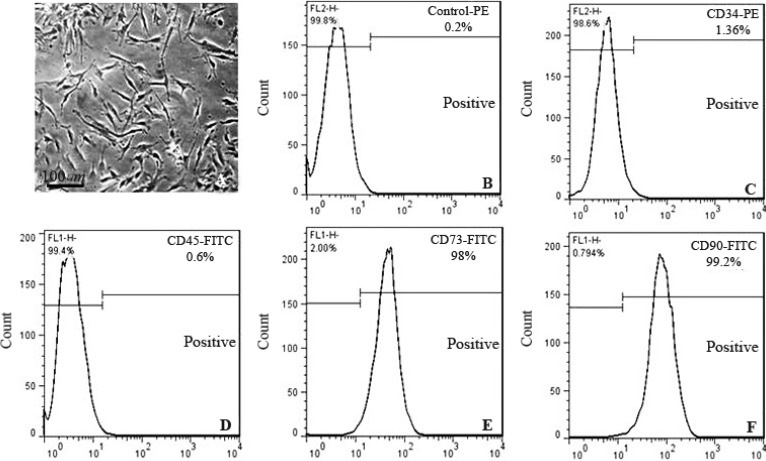
Flow cytometric analyses of the expression of various Bone-marrow-derived Mesenchymal Stromal Cells (BMSCs) markers. BMSCs were cultured for 4 passages and then the cells were lifted with trypsin free cell detachment buffer. Following incubation with the fluorescence dye conjugated antibodies specific for CD34, CD45, CD73 and CD90, the cells were analyzed by flow cytometry. Images are representative of 3–5 independent experiments. PE, phycoerythrin; FITC, fluorescein isothiocyanate.

Morphological observation of cultures by phase-contrast microscopy revealed that approximately all adherent cells exhibited typical fibroblast cell morphology (Figure 1A). To characterize these cells, the expression of a panel of markers, including CD34, CD45, CD73 and CD90, was examined by flow cytometry. The BMSCs were found to be negative for CD34 and CD45 and positive for the cell surface antigens CD73 and CD90 (Figures 1B–E).

### SDF-1 preconditioned BMSCs show increased homing in STZ injured pancreas

**Figure 2.**
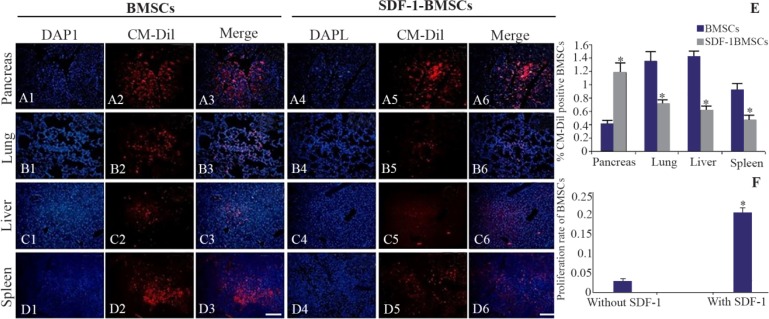
Homing of CM-Dil labelled Bone-marrow-derived Mesenchymal Stromal Cells (BMSCs) with or without stromal derived factor (SDF-1α) preconditioning following intravenous transplantation. The red fluorescent cells (CM-Dil labeled BMSCs) were observed in STZ-injured pancreas (A1–A3; without SDF-1, A4–A6; with SDF-1α) and lung (B1–B3; without SDF-1, B4–B6; with SDF-1 α), liver (C1–C3; without SDF-1α, C4–C6; with SDF-1α) and spleen (D1–D3; without SDF-1α, D4–D6; with SDF-1α). Quantification of fluorescence intensity of CM-Dil labeled cells (E) in bone-marrow-derived mesenchymal stromal cells without SDF-1(BMSCs) and with SDF-1α preconditioning (SDF-1α-BMSCs) transplanted groups by ImageJ(ImageJ for Windows, Version 1.50i). Proliferation rate of BMSCs with or without SDF-1α preconditioning by MTT assay (F). Data are expressed as means (n=3)±SD. Level of significance is p<0.05. Level of significance is p<0.05. Scale bar=100 *μm*.

Homing of CM-DiI labelled BMSCs with or without SDF-1α preconditioned were assessed 48 *hr* post intravenous transplantation. As shown in figure 2, the transplanted CM-DiI positive BMSCs exhibited homing specificity in pancreas, lung, liver, and spleen of injected mice. SDF-1α-preconditioned BMSCs displayed a significant (p≤0.05) increase in the homing of CM-DiI labeled BMSCs in pancreas, as compared with un-preconditioned BMSCs (Figures 2A1–A6 and E). Fluorescence intensity was quantified using ImageJ software (ImageJ for Windows, Version 1.50; NIH) (Figure 2E).

### Cell proliferative effect of SDF-1α

A relatively higher proliferation of BMSCs was observed in SDF-1α preconditioned BMSCs than un-preconditioned BMSCs group as measured by MTT assay. Our results showed that SDF-1α preconditioning induced a significant 7-fold increase in the cell proliferation of the BMSCs (Figure 2F).

### Flow cytometry analysis of CM-DiI positive BMSCs

**Figure 3.**
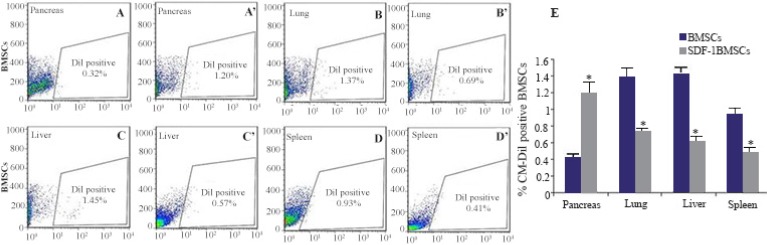
Bone-marrow-derived Mesenchymal Stromal Cells (BMSCs) distribution, with or without stromal derived factor (SDF-1α) preconditioning, within various organs after intravenous injection. Forty eight hours after intravenous transplantation, mice were sacrificed and organs, including pancreas, lung, liver and spleen, were harvested for BMSCs (A, B, C and D; without SDF-1α, A’, B’, C’ and D’; with SDF-1α) distribution, determined by CM-Dil fluorescence density under flow cytometry. Average data from the different organs of three mice are presented (E). Level of significance is p<0.05.

48 *hr* after transplantation of CM-DiI-labeled BM-SCs with or without SDF-1α preconditioning, STZ-induced diabetes mice were killed and single cell suspensions of pancreas, lung, liver and spleen were analyzed for the presence of CM-DiI -positive cells by flow cytometry. Results showed that BMSCs were found in the lung, liver and spleen but were barely detectable in the injured pancreas when transplanted without SDF-1α preconditioning. Homing numbers of BMSCs to the lung, liver and spleen were significantly reduced in the BMSCs with SDF-1α preconditioning, while a large number of BMSCs were detected in the injured pancreas (Figure 3).

### Effect of BMSCs transplantation in diabetic mice

**Figure 4.**
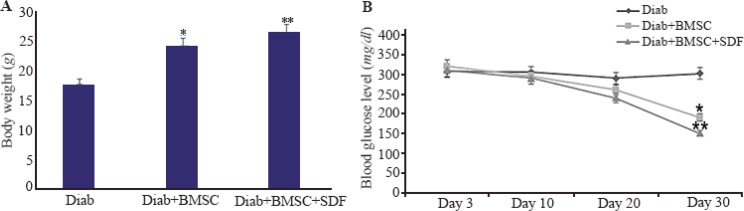
Body weight and blood glucose levels in streptozotocin induced diabetic and treated mice during the experiment. A, Effects of the transplantation of BMSCs with and without SDF-1α preconditioning on the body weight of mice with streptozotocin induced diabetes. B, Blood glucose levels in diabetic and treated groups at days 3, 10, 20 and 30 of study. Diab, Diabetic; BMSCs, bone-marrow-derived mesenchymal stromal cells; SDF-1α, stromal derived factor.*, Diab+BMSC group *vs*. diab group. **, Diab+BMSC+SDF-1α group *vs*. Diab+BMSC group. All values are mean±SD. Level of significance is p<0.05.

All mice fulfilled the criteria of diabetes after STZ as defined by an increase in blood glucose level more than 250 *mg/dl* on multiple occasions. Generally, the body weight was significantly increased in Diab+BMSC and Diab+BMSCs+SDF-1α animals compared to diabetic groups. However, following 30 days of the treatment, the body weight of mice in BMSC group was reduced as compared with BMSC+SDF-1α group. But during the whole study period, the body weight of BMSCs and BMSC+SDF-1α groups remained higher in comparison to diabetic mice (Figure 4A). Administration of BMSCs with or without SDF-1α preconditioning caused a significant decrease in blood glucose level on days 10, 20 and 30 in the experimental group compared with STZ induced diabetic group (p<0.001). However, the mice in group BMSC+SDF-1α showed reduced blood glucose level following 30 days of the treatment, compared with group BMSCs (Figure 4B).

### Fate of transplanted BMSCs

**Figure 5.**
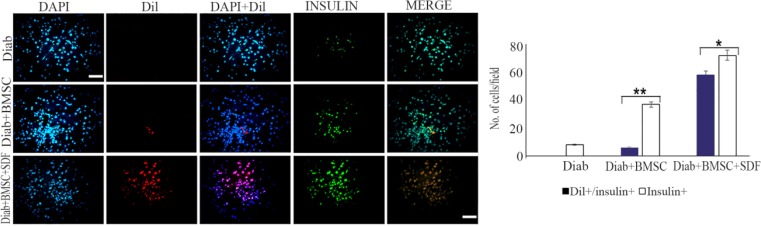
Bone-marrow-derived Mesenchymal Stromal Cells (BMSCs) with or without stromal derived factor (SDF-1α) preconditioning contribute to regenerate β-cells. Fluorescence microscopy images from pancreas of STZ-induced diabetic mice at 30 days after BMSCs transplantation. Notes: Column (DAPI), showing the blue fluorescence from DAPI-stained nuclei. Column (Dil) showing the Dil labeled BMSCs transplanted. Column (DAPI/Dil), showing merged of DAPI and Dil figures. Column (INSULIN), showing green fluorescence from FITC secondary antibody for insulin antibody slides. Column (MERGE), showing merged of DAPI/Dil and insulin figures. Quantification of Insulin positive (Insulin+) and CM-Dil positive with or without Insulin positive (CM-Dil+/Insulin+) cells were counted under a fluorescence microscope (BX51; Olympus, Tokyo, Japan), was using a 40× objective and a grid overlay. Average cell densities were calculated as number of cells per field. Level of significance is p<0.05. BMSCs, bone-marrow-derived mesenchymal stromal cells; DAPI, 4′, 6-diamidino-2-phenylindole; Dil, CM-Dil; FITC, fluorescein isothiocyanate. Scale bar=100 *μm*. ** p<0.001 versus control; * p<0.05 versus Diab+BMSC. Level of significance is p<0.05.

To define the contribution of host pancreatic cells versus that of transplanted cells to the process of regeneration, differentiation of transplanted cells in the pancreases of STZ-induced diabetic mice was studied. Transplanted BMSCs were detected in the host pancreas by their CM-DiI fluorescence. Immunofluorescent staining for insulin indicated that the vast majority of transplanted unpreconditioned BMSCs remained in an undifferentiated state. Moreover, there was further differentiation of transplanted preconditioned BMSCs, when examined in pancreas 30 days post transplantation (Figure 5).

### Histopathological findings

**Figure 6.**
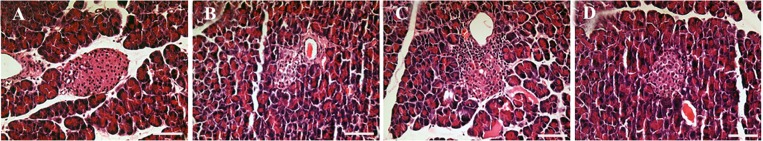
Histology of pancreas in normal, streptozotocin, Bone-marrow-derived Mesenchymal Stromal Cells (BMSCs) with or without stromal derived factor (SDF-1α) preconditioning following intravenous transplantation in diabetic mice. Changes of the pancreas histological structure in different treated groups were examined using hematoxylin and eosin staining: (A) pancreas of normal mice showing normal histological structure. (B) STZ-treated diabetic group showing atrophied islets of Langerhans. (C) STZ-diabetic mice treated with the BMSCs without SDF-1α preconditioning, showing remarkable recovery of pancreatic islets structure when compared with STZ-diabetic mice. (D) STZ-diabetic mice treated with BMSCs with SDF-1α preconditioning, showing significant changes in islet structure when compared with STZ-diabetic mice treated with the BMSCs without SDF-1α preconditioning. Scale bar=100 *μm*.

The effect of BMSCs on the histological structure of the pancreas was investigated. Pancreases of normal and treated mice were harvested at the endpoint of the study and stained with H and E. Pancreatic sections from normal mice exhibited islets of Langerhans cells, and contained lightly stained acidophilic cells arranged in branching and anastomosing cords (Figure 6A). Sections from STZ-diabetic mice displayed damaged islets, represented by cytoplasmic vacuolations, pyknotic nuclei and atrophied islets of Langerhans (Figure 6B). Sections from STZ-diabetic mice that received BMSCs without SDF-1 preconditioning exhibited remarkable recovery of pancreatic islets structure when compared with STZ-diabetic mice (Figure 6C). Sections from STZ-diabetic mice that received SDF-1α preconditioned BMSCs exhibited more severe islet alterations and recovery when compared with STZ-diabetic mice treated with the BMSCs without SDF-1 preconditioning (Figure 6D).

## Discussion

In the present study, it was found that SDF-1α preconditioning enhanced BMSCs proliferation *in vitro*. Next, it was demonstrated that SDF-1α preconditioning before transplantation improved BMSCs homing toward damaged pancreatic tissues after STZ-induced diabetes mellitus, which was accompanied by improved histological appearance and blood glucose control. It has been found that during intravenous transplantation of MSCs, there is a reduction of injected cells in the vasculature and on the other hand, mostly they were trapped in the organs like lungs and liver. So, in this study, homing of BMSCs was assessed in the lung, liver and spleen beside the organ of interest, pancreas 3. Nowadays, the well-established clinical experience in the field of hematology has encouraged the use of BMSCs in non-hematological diseases 17. The BMSCs have great interest as a promising cell source for regenerative medicine due to high expansion capacity, easy availability, and low immunogenicity 18. There are several routes of cell delivery for treatment of diabetes without affecting their viability and efficacy 19. Intravenous transplantation of BMSCs is an attractive option in the context of regenerative medicine as this may result in benefit for multiple complications and has the potential to improve glycemic control 20. However, the success of a vascular route for BMSC treatment may be limited by the low migration ability of the transplanted BMSCs into the lesion area 21. Therefore, the interventions that enhance the migration of BMSCs are the key to optimize BMSC therapy in diabetes 8.

Preconditioning is a powerful tool through which cells can be protected and is one intervention that is easily adaptable to the clinic for improving cell survival and long-term engraftment after transplantation 22. Transplantation of BMSCs preconditioned with different regimens, such as chemical drugs, cytokines and hypoxia has shown much better engraftment and efficacy in recent years 23. It has been extensively demonstrated that the SDF-1/CXCR4 axis displays a critical role in adhesion, migration and homing of the mobilized BMSCs towards the injured areas 13,23. CXCR4, the specific receptor of SDF-1α, was extensively expressed in BMSCs and is usually absent on the surface of culture-expanded MSCs 24. For unknown reasons, BMSCs tend to lose the expression of homing molecules, such as CXCR4, during *in vitro* expansion 23.

In this study, there were notable differences in blood glucose level between the groups administered with BMSCs with SDF-1α preconditioning and BMSCs without SDF-1α preconditioning, which are believed to result from the fact that proliferation rate of BMSCs is increased by SDF-1α preconditioning. Few authors claim that SDF-1α preconditioning not only changes the migratory capacities of BMSCs but also plays an important role in BMSCs survival because BMSCs pretreated with SDF-1α exhibited significantly improved survival and proliferation 19,23–25. Our results reported in the present study supported the proposal that SDF-1α preconditioning promotes BMSCs proliferation and enhanced BMSCs homing toward damaged tissues. Similar to our data, in the animal model of ischemic myocardium, majority of the systemic transplanted MSCs cells were entrapped in the liver, lungs, and only a slight fraction home in the site of injury. But following CXCR4 gene modifications, homing of MSCs was significantly higher in ischemic myocardium, compared to lung, liver and spleen 3. In addition, it was demonstrated that intravenous administration of SDF-1α preconditioned BMSCs is sufficient to decrease blood glucose levels in a more physiologic range with marked improvement in body weight loss in the treated diabetic mice rather than unpreconditioned BMSCs. Previous studies suggested that BMSCs have the ability of reducing glucose levels in animals or subjects with type1 diabetes 5,15,20,25. To date, the precise mechanisms that underlie the functional interaction between BMSCs and pancreas are unclear.

Basically, two hypotheses have been postulated that try to explain the underlying mechanism of the therapeutic effect of BMSCs on diabetes: direct differentiation of BMSCs into functionally competent β-cells or indirectly through paracrine-mediated mechanisms 20,26. Many researchers believe that the beneficial effects provided by BMSCs transplantation would be primarily based on their paracrine activity by releasing trophic and immunomodulatory factors 27. Previous experiments had shown that expression of antiapoptotic, anti-inflammatory, and proangiogenic molecules by transplanted BMSCs might represent mechanisms that induce and accelerate pancreatic repair 28.

Although a large number of studies suggest that the mechanism is predominantly paracrine-mediated, the role of BMSCs differentiation into β-cells cannot be fully excluded 26. It has been demonstrated that Mesenchymal Stem Cell (MSCs) have the ability to differentiate into insulin-producing cells and secrete insulin in response to glucose and reverse the hyperglycemia in diabetic mice 25. A number of authors have described the capability of MSC to differentiate into insulin secreting cells *in vivo* 5,15,20,25,26. It has been shown that BMSCs could form islet-like structures, and phenotypically and functionally transdifferentiate to insulin-producing cells 29,30. With intravenous injection, the limited number of transplanted BMSCs participating in pancreas regeneration and the small amount of insulin produced by these cells seemed to be inadequate to maintain euglycemia 26. However, the exact mechanism by which BMSCs induced normoglycemia in this study is not clear yet.

## Conclusion

Our results showed the effectiveness of SDF-1α preconditioning in BMSCs transplantation of STZ-induced diabetes mice which might be achieved through improvement of BMSCs homing into the injured pancreas.
